# Obeticholic acid protects against hepatocyte death and liver fibrosis in a murine model of nonalcoholic steatohepatitis

**DOI:** 10.1038/s41598-018-26383-8

**Published:** 2018-05-25

**Authors:** Toshihiro Goto, Michiko Itoh, Takayoshi Suganami, Sayaka Kanai, Ibuki Shirakawa, Takeru Sakai, Masahiro Asakawa, Toshihiro Yoneyama, Toshihiro Kai, Yoshihiro Ogawa

**Affiliations:** 1 Department of Molecular Endocrinology and Metabolism, Graduate School of Medical and Dental Sciences, Tokyo Medical and Dental University, Tokyo, Japan; 2 Specialty Medicine Group, Drug Development Research Laboratories, Sumitomo Dainippon Pharma. Co., Ltd, Osaka, Japan; 3 Department of Organ Network and Metabolism, Graduate School of Medical and Dental Sciences, Tokyo Medical and Dental University, Tokyo, Japan; 4 Department of Molecular Medicine and Metabolism, Research Institute of Environmental Medicine, Nagoya University, Nagoya, Japan; 5 Department of Molecular and Cellular Metabolism, Graduate School of Medical and Dental Sciences, Tokyo Medical and Dental University, Tokyo, Japan; 6 Omics Group, Genomic Science Laboratories, Sumitomo Dainippon Pharma. Co., Ltd, Osaka, Japan; 7 Department of Medicine and Bioregulatory Science, Graduate School of Medical Sciences, Kyushu University, Fukuoka, Japan; 8 Japan Agency for Medical Research and Development, CREST, Tokyo, Japan

## Abstract

Accumulating evidence has suggested that farnesoid X receptor (FXR) agonists, such as obeticholic acid (OCA) are therapeutically useful for non-alcoholic steatohepatitis (NASH). However, it is still unclear how FXR agonists protect against NASH and which cell type is the main target of FXR agonists. In this study, we examined the effects of OCA on the development of NASH using melanocortin 4 receptor-deficient (MC4R-KO) mice that progressively developed hepatic steatosis and NASH on Western diet (WD). Treatment with OCA effectively prevented chronic inflammation and liver fibrosis in WD-fed MC4R-KO mice with only marginal effect on body weight and hepatic steatosis. Hepatic crown-like structure (hCLS) is a unique histological structure characteristic of NASH, which triggers hepatocyte death-induced interstitial fibrosis. Intriguingly, treatment with OCA markedly reduced hCLS formation even after MC4R-KO mice developed NASH, thereby inhibiting the progression of liver fibrosis. As its mechanism of action, OCA suppressed metabolic stress-induced p53 activation and cell death in hepatocytes. Our findings in this study highlight the role of FXR in hepatocytes in the pathogenesis of NASH. Collectively, this study demonstrates the anti-fibrotic effect of OCA in a murine model of NASH with obesity and insulin resistance, which suggests the clinical implication for human NASH.

## Introduction

Non-alcoholic fatty liver disease (NAFLD) is recognized worldwide as the most prevalent chronic liver disease and is frequently accompanied with obesity and type 2 diabetes mellitus^1,2^. NAFLD encompasses a wide spectrum of liver impairments ranging from simple steatosis to nonalcoholic steatohepatitis (NASH), the latter of which eventually proceeds to cirrhosis and hepatocellular carcinoma^3^. NASH is characterized by hepatocyte death, inflammation and varying degree of interstitial fibrosis^4^, although its precise cause is still unclear. Among several histological features, metabolic stress-induced hepatocyte death is considered a key event during the disease progression from simple steatosis to NASH. Although accumulating evidence is suggesting that lifestyle modification, including diet and physical activity may improve central obesity and insulin resistance and therefore manage NAFLD/NASH, it is necessary to expand the currently limited pharmacological interventions to therapies with superior efficacy^5^.

Farnesoid X receptor (FXR) is a member of the nuclear receptor superfamily expressed in the liver, kidney, intestine and adrenal glands^6,7^. In addition to regulation of bile acid (BA) synthesis^8^, several lines of evidence have suggested that FXR plays a role in the pathophysiology of NAFLD/NASH^9^. For instance, aged FXR-deficient mice are reported to develop NASH-like liver pathology^10^. Moreover, FXR agonists, such as obeticholic acid (OCA, 6α-ethyl-chenodeoxycholic acid), a semi-synthetic variant of chenodeoxycholic acid, have been shown to produce beneficial effects on cholestasis^11^, steatosis^12–15^, and fibrosis in experimental models^14–17^, whereas the effect on obesity-associated liver fibrosis remains to be fully elucidated^12^. It is also known that OCA produces potent anti-inflammatory and anti-fibrotic effects in cultured hepatic stellate cells (HSCs) and macrophages^17,18^. Moreover, several studies support the clinical relevance of OCA, among which a multicenter, randomized, placebo-controlled trial (the FLINT study) demonstrates that treatment with OCA improves the biochemical and histological features of NASH^19–21^. However, it is still unclear how OCA protects against NASH and which cell type is the main target of OCA, since stromal cells, such as HSCs and macrophages as well as parenchymal hepatocytes express FXR.

Animal models are indispensable for “proof-of-mechanism” studies that support the introduction of novel therapeutic agent into clinical practice. Recently, we have reported a murine NASH model using melanocortin 4 receptor-deficient (MC4R-KO) mice^22^. MC4R is a seven-transmembrane G protein-coupled receptor predominantly expressed in the brain and regulates energy homeostasis^23^. In addition to the known phenotypes, such as obesity and insulin resistance, MC4R-KO mice on high-fat diet or Western diet (WD) progressively exhibit hepatic steatosis, NASH and multiple liver tumors. Using this model, we have identified a unique histological structure termed “hepatic crown-like structure (hCLS)”, in which macrophages surround dead or dying hepatocytes with large lipid droplets, thereby activating HSCs to induce interstitial fibrosis^24^. Of note, hCLS observed in human NASH is rarely seen in viral hepatitis-induced liver fibrosis. Since hCLS provides a local microenvironment in steatotic liver where cell death-triggered stromal cellular responses drive profibrotic mechanisms, hCLS is a histological feature by which target molecules/cells as well as therapeutic efficacy can be evaluated^25,26^. These findings led us to examine the effect of OCA on the development of NASH and its potential molecular mechanism.

We found in this study that treatment with OCA markedly suppresses hepatocyte death, hCLS formation and liver fibrosis with only marginal effects on body weight and hepatic steatosis. As a molecular mechanism, OCA suppressed metabolic stress-induced p53 activation and cell death in hepatocytes. Our findings highlight the role of FXR in hepatocytes in the pathogenesis of NASH and demonstrate the anti-fibrotic effect of OCA in a unique murine model of NASH with obesity and insulin resistance.

## Results

### Preventive effect of OCA on NASH in MC4R-KO mice

**Figure 1:**
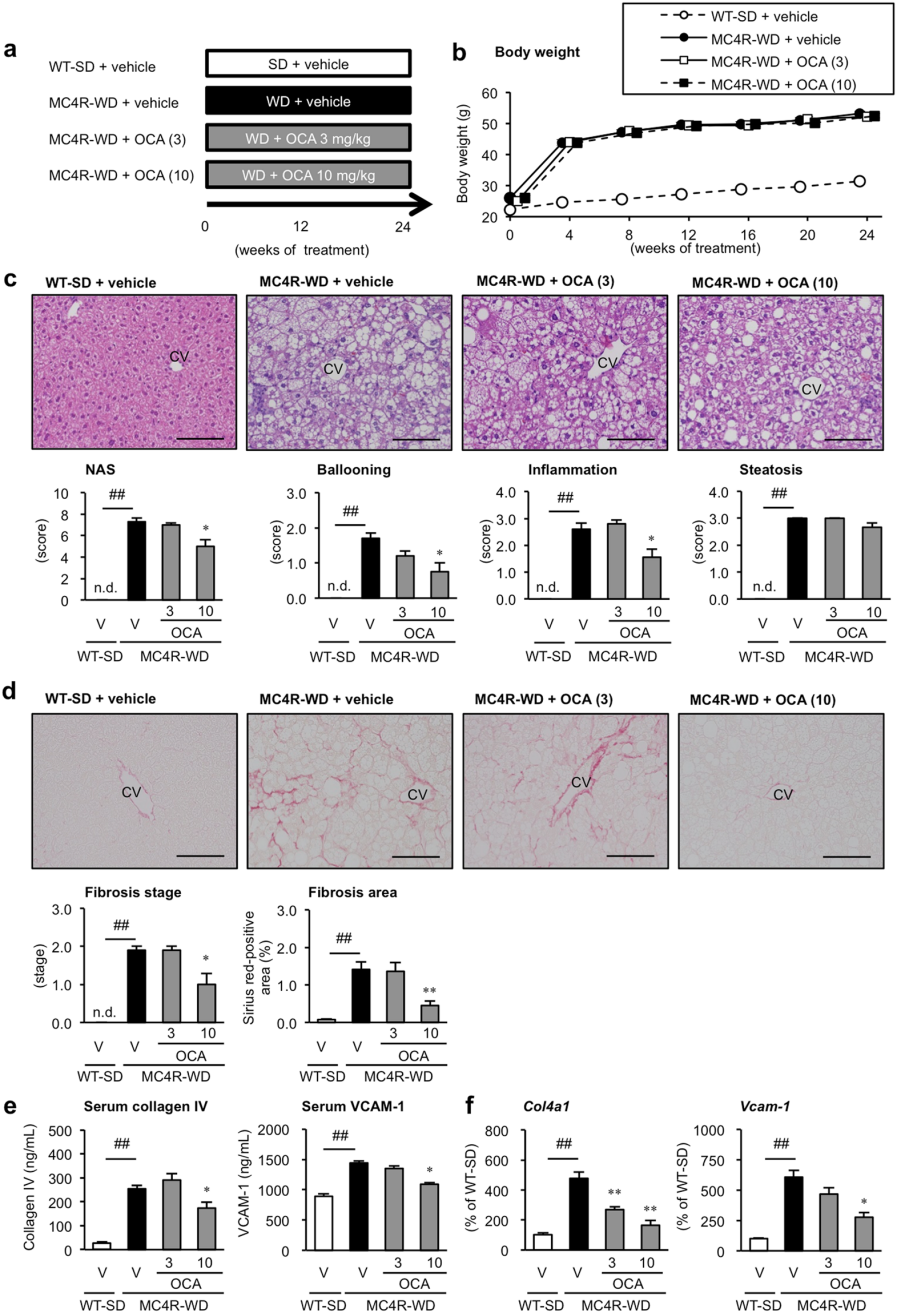
Preventive effect of OCA on the development of NASH in MC4R-KO mice. (**a**) Experimental protocol for examination of the preventive effect of obeticholic acid (OCA) on the development of NASH in melanocotin-4 receptor deficient (MC4R-KO) mice fed Western diet (MC4R-WD). Vehicle, 0.5% carboxymethyl cellulose (CMC). Wild type (WT) mice on standard diet (WT-SD) treated with the vehicle were used as the control group. (**b**) Growth curve of WT and MC4R-KO mice. Open circle, WT-SD treated with the vehicle (*n* = 7); closed circle, MC4R-WD treated with the vehicle (*n* = 10); open and closed square, MC4R-WD treated with OCA at 3 mg/kg (*n* = 10) and 10 mg/kg (*n* = 9), respectively. (**c**) Hematoxylin and eosin staining of the liver after vehicle or OCA treatment. Histological analysis using non-alcoholic fatty liver disease (NAFLD) activity score (NAS). (**d**) Evaluation of liver fibrosis using Sirius red staining. (**e**) Serum concentrations of collagen type IV and soluble vascular cell adhesion molecule-1 (VCAM-1). (**f**) Hepatic mRNA expression levels of collagen type IV and VCAM-1. Scale bars, 100 μm. CV, central veins. V, Vehicle, n.d., not detected. ^##^*p* < 0.01; ^*^p < 0.05, ^**^p < 0.01 vs. vehicle-treated MC4R-WD group.

We first examined the preventive effect of OCA on the development of NASH in a murine model of NASH using MC4R-KO mice. OCA (3 or 10 mg/kg, p.o.) or its vehicle was administered daily for 24 weeks to MC4R-KO mice fed WD (MC4R-WD) and wild type mice fed standard diet (WT-SD) (Fig. 1a). Long-term treatment with OCA had no effect on body weight and adipose tissue weight in MC4R-KO mice (Fig. 1b, Supplementary Fig. S1a). However, OCA at 10 mg/kg markedly decreased liver weight (Supplementary Fig. S1a) and reduced serum concentrations of alanine aminotransferase (ALT), aspartate aminotransferase (AST), total cholesterol (TC) and BA (Table 1, Supplementary Table S2) as well as hepatic content of TC and BA in MC4R-KO mice (Supplementary Fig. S1b, Supplementary Table S2). On the other hand, treatment with OCA did not affect serum concentrations of triglyceride (TG), non-esterified fatty acid (NEFA), blood glucose (BG) and insulin (Table 1), and hepatic content of TG and secondary BAs (deoxycholic acid and taurodeoxycholic acid) (Supplementary Fig. S1b, Supplementary Table S3).

**Table 1:** Preventive effect of OCA on serological parameters of MC4R-KO fed WD for 24 weeks.

	WT-SD	MC4R-WD		
	Vehicle	Vehicle	OCA (3)	OCA (10)
BG (*ad lib*, mg/dL)	126.1 ± 3.6	146.1 ± 8.7	119.1 ± 5.5^*^	138.4 ± 9.2
Insulin (*ad lib*, ng/ml)	0.8 ± 0.2	31.7 ± 6.5^##^	35.9 ± 6.7	50.7 ± 6.8
TC (mg/dL)	88.4 ± 3.8	337.0 ± 16.0^##^	300.0 ± 15.0	232.0 ± 8.5^**^
TG (mg/dL)	112.4 ± 12.6	58.0 ± 6.2^##^	46.2 ± 8.6	47.1 ± 4.7
NEFA (mEq/L)	1.26 ± 0.06	1.07 ± 0.05^#^	1.01 ± 0.06	0.94 ± 0.06
ALT (U/L)	29.2 ± 1.0	439.6 ± 58.3^##^	412.8 ± 43.8	160.9 ± 31.2^**^
AST (U/L)	157.6 ± 13.8	381.2 ± 33.9^##^	384.6 ± 39.5	210.0 ± 25.2^**^

WT, wildtype mice; MC4R-KO, melanocortin 4 receptor deficient mice; SD, standard diet; WD, Western diet; OCA, obeticholic acid; BG, blood glucose; TC, total cholesterol; TG, triglyceride; NEFA, non-esterified fatty acid; ALT, alanine aminotransferase; AST, aspartate transaminase. Data are expressed as the mean ± SE. *n* = 6–10. ^#^*p* < 0.05, ^##^*p* < 0.01 vs. WT-SD group, ^*^p < 0.05, ^**^p < 0.01 vs. vehicle-treated MC4R-WD group.

At 24 weeks of WD feeding, MC4R-KO mice exhibited histologic features characteristic of NASH, including micro- and macrovesicular steatosis, ballooning degeneration, massive infiltration of inflammatory cells and pericellular fibrosis (Fig. 1c,d)^22^. Although no change in steatosis score was observed, the scores of inflammation and ballooning degeneration decreased following treatment with OCA at 10 mg/kg, as indicated by a significant reduction in NAFLD activity score (NAS) in OCA (10 mg/kg)-treated MC4R-KO mice relative to the vehicle-treated group (Fig. 1c). Notably, OCA markedly ameliorated liver fibrosis (Fig. 1d) and significantly decreased serum concentrations of collagen type IV and soluble vascular cell adhesion molecule-1 (VCAM-1), two noninvasive markers clinically useful for the diagnosis of NASH and NASH-related fibrosis (Fig. 1e)^27^. mRNA levels of these two markers in the liver were also markedly reduced following treatment with OCA (Fig. 1f). On the other hand, VCAM-1 serum concentrations and hepatic mRNA levels were increased in MC4R-KO mice at 20 weeks of WD feeding, although no apparent change was observed at 4–5 weeks (Supplementary Fig. S1c,d). These results indicate that OCA effectively prevents the development of NASH in MC4R-KO mice.

### Treatment with OCA inhibits chronic inflammation-induced liver fibrosis in MC4R-KO mice

**Figure 2:**
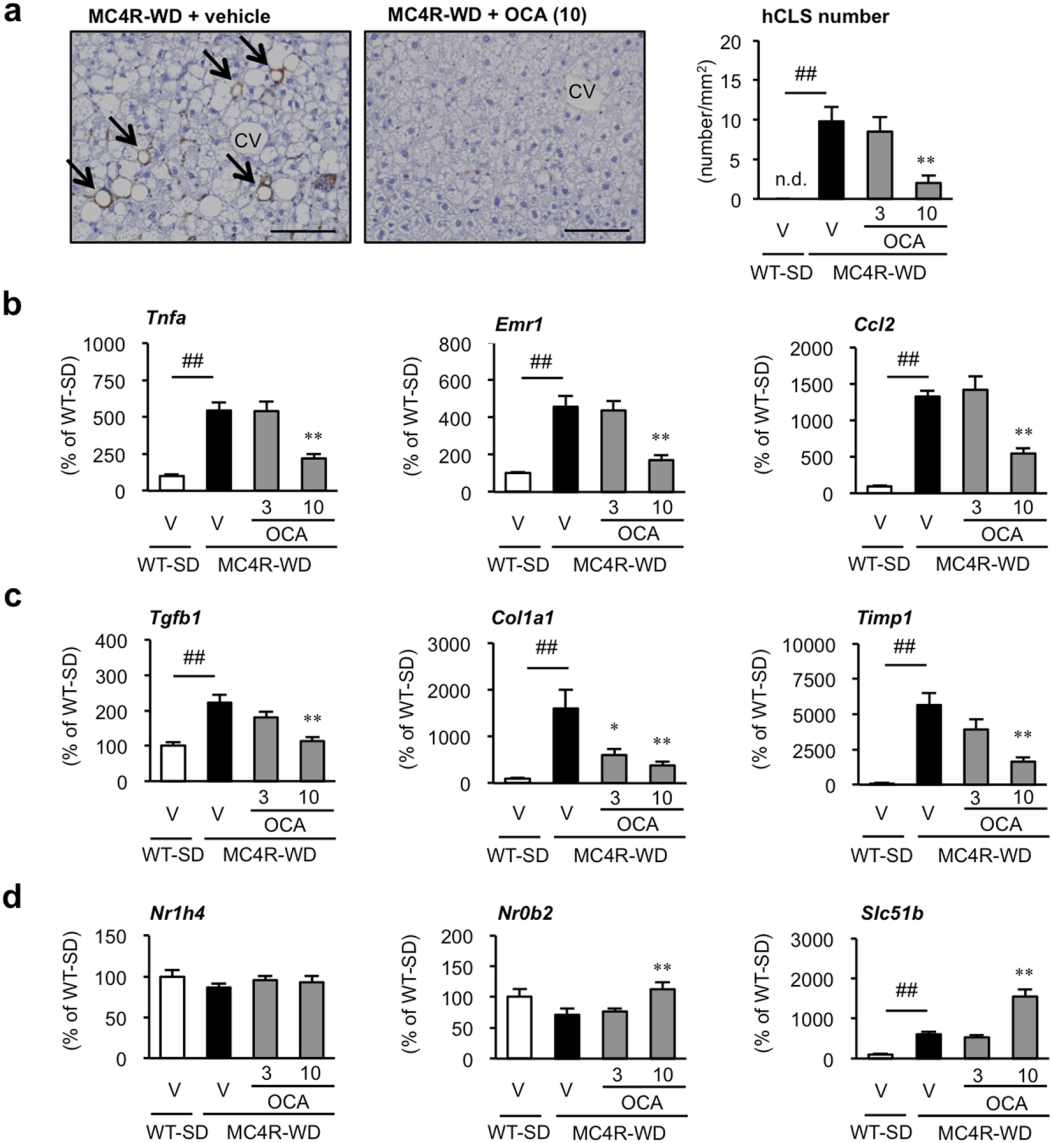
Treatment with OCA inhibits hCLS formation during the development of NASH. (**a**) F4/80 immunostaining of the liver after treatment with OCA for 24 weeks. Arrows indicate hepatic crown-like structures (hCLS). Scale bars, 100 μm. CV, central veins. V, Vehicle, n.d., not detected. Hepatic mRNA expression levels of genes related to (**b**) inflammatory markers (*Tnfa*, *Emr1* and *Ccl2*) and (**c**) fibrogenic factors (*Tgfb1*, *Col1a1* and *Timp1*). (**d**) Hepatic mRNA expression levels of farnesoid X receptor (FXR, *Nr1h4*) and FXR-target genes (*Nr0b2* and *Slc51b*). ^##^*p* < 0.01; ^**^p < 0.01 vs. vehicle-treated MC4R-WD group. Vehicle-treated WT-SD group, *n* = 7; vehicle-treated MC4R-WD group, *n* = 10; 3 and 10 mg/kg of OCA-treated MC4R-WD group, *n* = 10 and *n* = 9, respectively.

Next, we investigated whether treatment with OCA affects hCLS formation in MC4R-KO mice, since hCLS serves as the source of chronic inflammation-induced fibrosis during the progression from simple steatosis to NASH^24,26^. Immunostaining for F4/80, a representative macrophage marker, revealed a marked decrease in hCLS formation in OCA-treated MC4R-KO mice (Fig. 2a). Consistent with this observation, hepatic mRNA expression of genes related to inflammation (tumor necrosis factor α [*Tnfa*], F4/80 [*Emr1*] and C-C motif chemokine 2 [*Ccl2*]) and fibrogenesis (transforming growth factor-β [*Tgfb1*], collagen type I [*Col1a1*] and tissue inhibitor of metalloproteinase-1 [*Timp1*]) was significantly suppressed in OCA-treated MC4R-KO mice relative to the vehicle-treated group (Fig. 2b,c). Although mRNA expression of FXR (*Nr1h4*) was unchanged, OCA treatment to MC4R-KO mice dose-dependently increased mRNA expression of small heterodimer partner (SHP, *Nr0b2*), organic solute transporter β (OST-β, *Slc51b*), and ATP-binding cassette subfamily B member 11 (*Abcb11*), and decreased that of sterol regulatory element binding protein 1c (*Srebf1c*) (Fig. 2d and Supplementary Fig. S2). In addition, OCA treatment suppressed expression of cytochrome P450 7A1 (*Cyp7a1*) and cytochrome P450 8b1 (*Cyp8b1*), although the changes did not reach a statistical significance (Supplementary Fig. S2). Taken together, these findings suggest that OCA effectively suppresses the formation of hCLS, thereby inhibiting chronic inflammation-induced liver fibrosis in MC4R-KO mice.

### Treatment with OCA suppresses p53 pathway and cell death during NASH development

**Figure 3:**
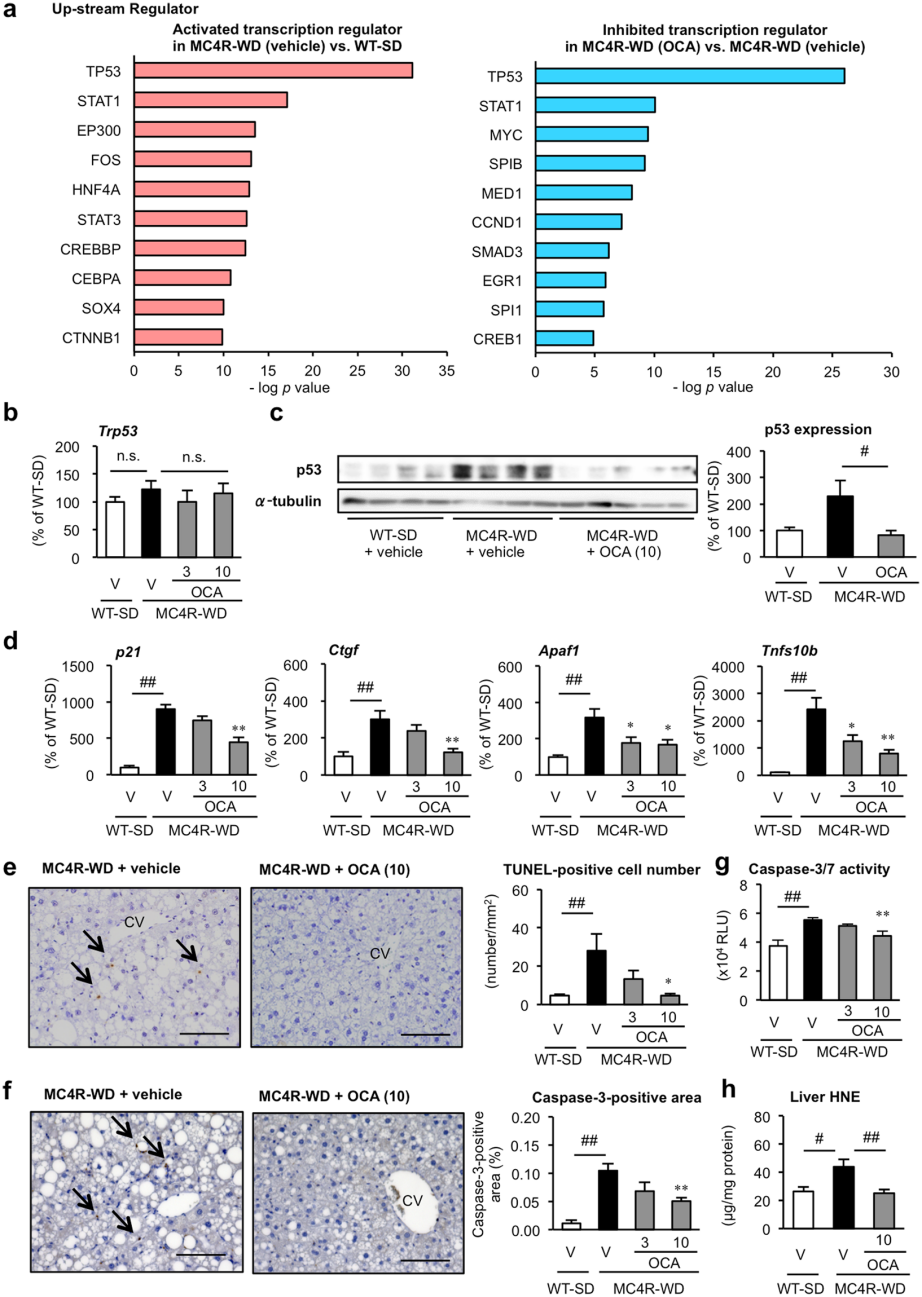
Treatment with OCA down-regulates p53 pathway and suppresses cell death in NASH. (**a**) Ingenuity Pathway Analysis of top 10 activated and inhibited up-stream transcription regulators in the vehicle-treated WT-SD group vs. vehicle-treated MC4R-WD group and vehicle-treated MC4R-WD group vs. OCA (10 mg/kg)-treated MC4R-WD group, respectively. Hepatic expression levels of (**b**) p53 mRNA and (**c**) p53 protein in the liver. (**d**) Hepatic mRNA expression levels of p53-regulated genes (*p21*, *Ctgf*, *Apaf1* and *Tnfs10*) after 24 weeks of OCA treatment. (**e**) TdT mediated dUTP-biotin nick end labeling (TUNEL) staining of the liver. Arrowheads indicate TUNEL-positive cells. (**f**) Representative images of caspase-3 immunstaining of the liver and quantification of caspase-3-positive area. Scale bars, 100 μm. CV, central veins. (**g**) Caspase-3/7 activity in the liver. (**h**) Hepatic levels of 4-hydroxynonenal, an oxidative stress-induced product of lipid peroxidation. ^#^*p* < 0.05, ^##^*p* < 0.01; ^**^p < 0.01 vs. the vehicle-treated MC4R-WD group. n.s., not significant. Vehicle-treated WT-SD group, *n* = 7; vehicle-treated MC4R-WD group, *n* = 10; 3 and 10 mg/kg OCA-treated MC4R-WD group, *n* = 10 and *n* = 9, respectively.

To clarify the preventive effect of OCA on NASH development, we performed a microarray analysis of livers from the vehicle- and OCA (10 mg/kg)-treated MC4R-KO mice. Ingenuity Pathway Analysis revealed that several signaling pathways related to inflammation and fibrosis were promoted in WD-fed MC4R-KO mice relative to SD-fed WT mice (Supplementary Fig. S3a). Treatment with OCA inhibited these pathways (Supplementary Fig. S3b) and, in parallel, improved histological scores (Fig. 1c,d). Further analysis of activated- and inhibited-transcriptional regulators using the microarray data revealed a particularly high activation of p53 in WD-fed MC4R-KO mice relative to SD-fed WT mice (Fig. 3a, *left*). This activation of p53 was markedly suppressed by treatment with OCA (Fig. 3a, *right*). Although no appreciable difference in p53 mRNA expression was observed between the groups (Fig. 3b), p53 protein levels, as well as mRNA expression of p53-regulated genes were markedly increased in MC4R-KO mice fed WD (Fig. 3c,d). These changes were significantly inhibited by treatment with OCA (Fig. 3c,d). Intriguingly, there was no appreciable difference in ubiquitin ligase MDM2 protein levels between vehicle-treated and OCA-treated MC4R-KO mice, although *Mdm2* mRNA levels were lower in OCA-treated MC4R-KO mice (Supplementary Fig. S3a,b). Western blot analysis revealed that phosphorylation at Ser15 and acetylation at Lys379 were significantly increased in the livers from WD-fed MC4R-KO mice compared to SD-fed wildtype mice, whereas Lys379 acetylation levels were significantly suppressed by OCA treatment (Supplementary Fig. S3c,d).

Since there is substantial evidence indicating the involvement of p53 and hepatocyte death in liver fibrosis in humans and rodents^28–30^, we next evaluated the effect of OCA on cell death using TdT mediated dUTP-biotin nick end labeling (TUNEL) staining. The number of TUNEL-positive cells was significantly increased in MC4R-KO mice (Fig. 3e), as reported previously^26^. This increase was reversed by treatment with OCA (Fig. 3e). We confirmed the effect of OCA on cell death using caspase-3 immunostaining and caspase-3/7 activity assay (Fig. 3f,g). In addition, OCA treatment suppressed hepatic levels of 4-hydroxynonenal, an oxidative stress-induced product of lipid peroxidation (Fig. 3h). Collectively, these findings suggest that OCA suppresses p53 activation and cell death during the development of NASH in MC4R-KO mice.

### Treatment with OCA prevents activation of p53 pathway in hepatocytes

**Figure 4:**
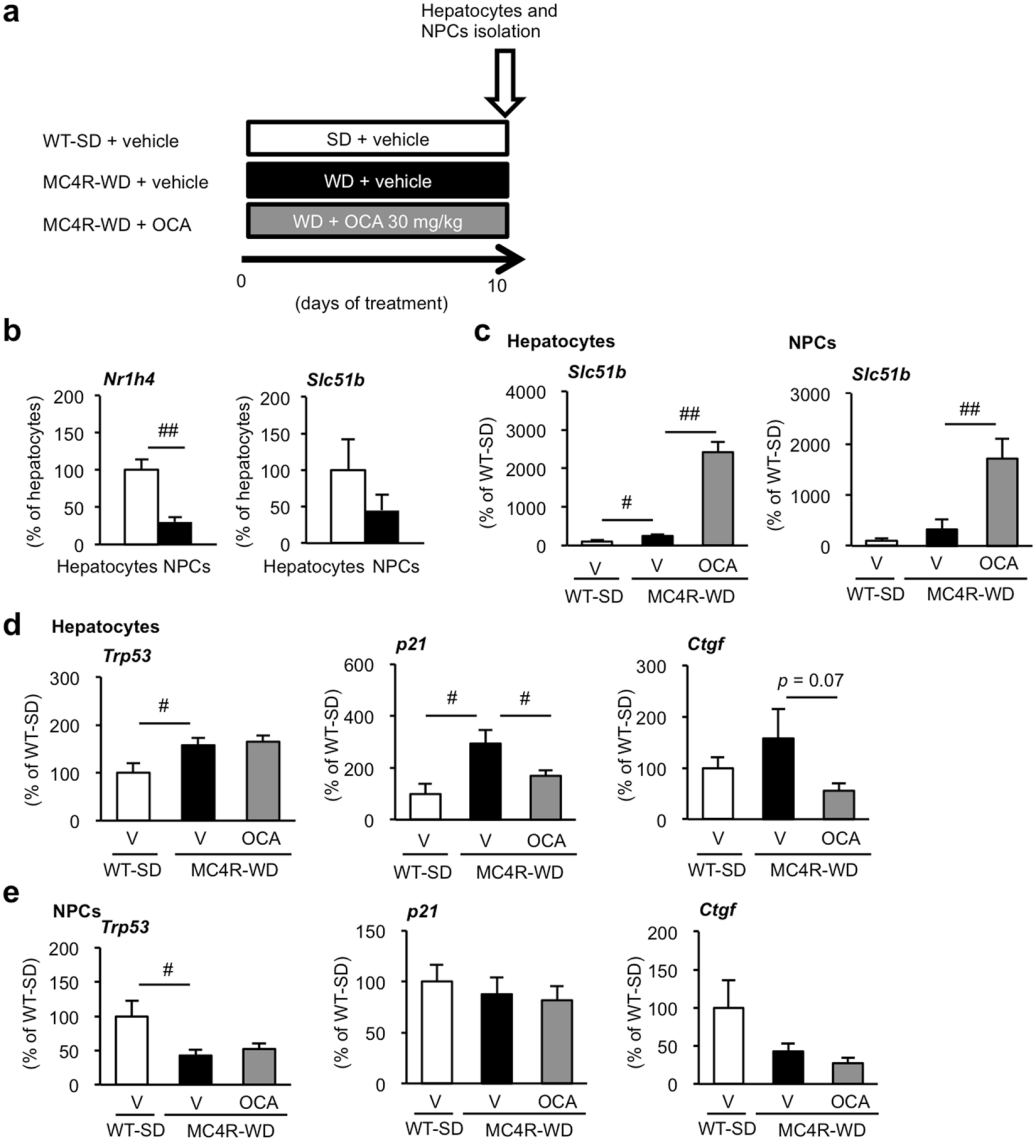
Treatment with OCA prevents activation of p53 pathway in hepatocytes of MC4R-KO mice. (**a**) Experimental protocol for evaluation of the preventive effect of OCA on p53 activation in isolated hepatocytes and non-parenchymal cells (NPCs) of short-term WD-fed MC4R-KO mice. mRNA expression levels of FXR (*Nr1h4*) and FXR-regulated organic solute transporter β (Ost-β, *Slc51b*) in hepatocytes and NPCs of WT mice (**b**), and mRNA expression levels of *Slc51b* in each fraction of SD-fed WT and WD-fed MC4R-KO mice treated with vehicle or OCA for 10 days (**c**). mRNA expression of *Trp53* and p53-regulated genes (*p21* and *Ctgf*) in (**d**) hepatocytes and (**e**) NPCs after treatment with OCA. V, Vehicle. ^#^*p* < 0.05, ^##^*p* < 0.01. Vehicle-treated WT-SD group, *n* = 4–5; vehicle-treated MC4R-WD group, *n* = 7–8; OCA-treated MC4R-WD group, *n* = 8–9.

We next examined whether treatment with OCA suppresses p53 activation in hepatocytes or non-parenchymal cells (NPCs) in WD-fed MC4R-KO mice. MC4R-KO mice following 10 days of WD feeding were treated with OCA (30 mg/kg) or vehicle, and their hepatocytes and NPCs were isolated (Fig. 4a), at which the hepatic content of triglyceride in MC4R-KO mice reaches double of that in lean wildtype mice (data not shown). FXR and its regulated gene, OST-β, were predominantly expressed in hepatocytes as compared to NPCs (Fig. 4b). Treatment with OCA markedly increased OST-β mRNA expression in both hepatocytes and NPCs (Fig. 4c), suggesting FXR pathway is activated in multiple cell types in the liver. Although mRNA expression of *Trp53* and p53-regulated genes (cyclin-dependent kinase inhibitor 1 A [*p21*] and connective tissue growth factor [*Ctgf*]) was upregulated in hepatocytes of vehicle-treated MC4R-KO mice, OCA treatment significantly suppressed mRNA expression of p53-regulated genes (Fig. 4d). In contrast, there was no apparent change in mRNA expression of these genes in NPCs with OCA treatment (Fig. 4e). These findings suggest that treatment with OCA suppresses metabolic stress-induced activation of p53 pathway mainly in hepatocyte.

### OCA suppresses hepatocyte death and liver fibrosis in “inducible NASH model”

**Figure 5:**
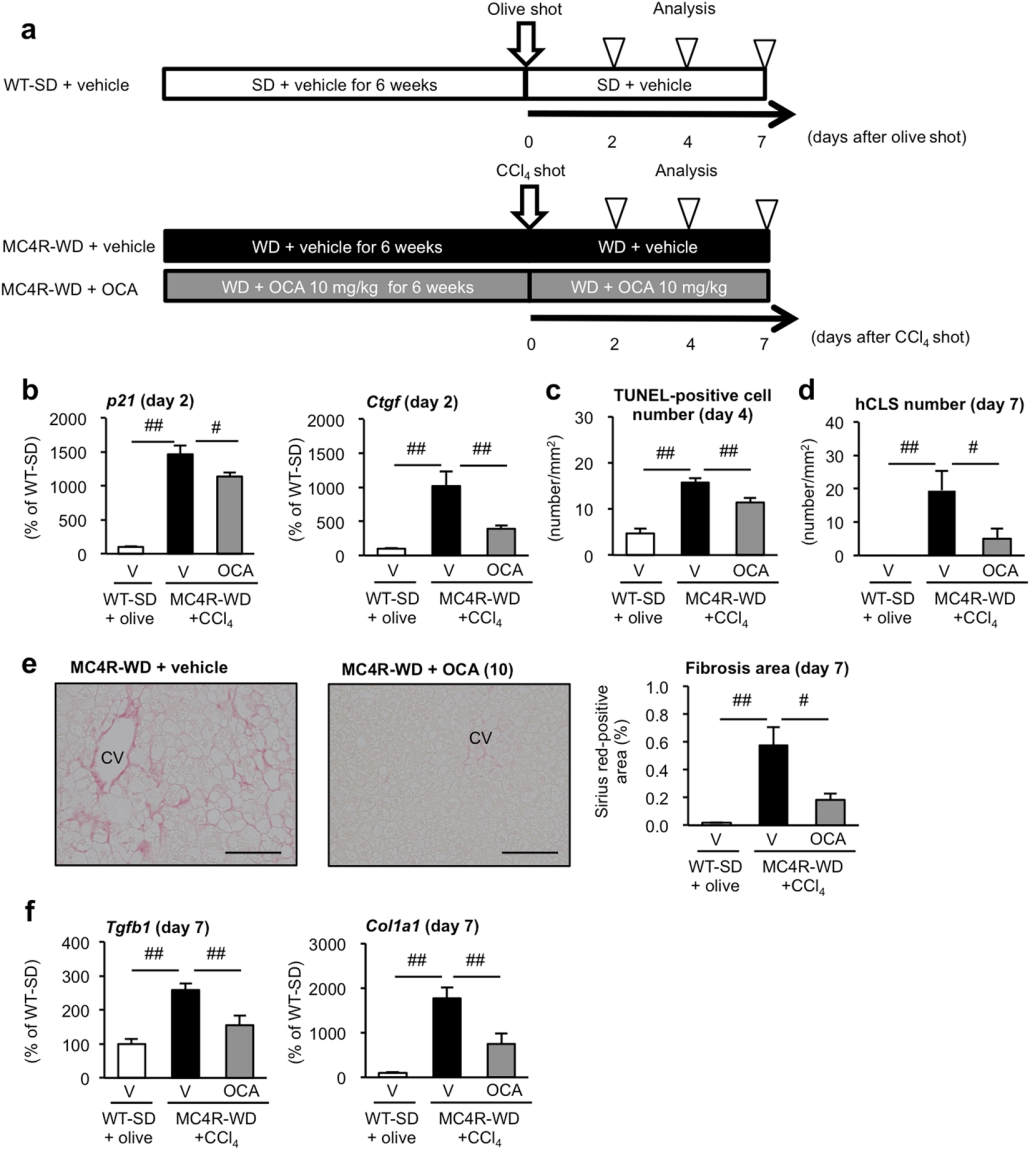
Treatment with OCA prevents hCLS formation and liver fibrosis in “inducible NASH model”. (**a**) Experimental protocol for examination of the preventive effect of OCA on hCLS formation and liver fibrosis in the inducible NASH model. (**b**) Hepatic mRNA expression levels of genes related to p53-regulated genes (*p21* and *Ctgf*) 2 days after CCl_4_ injection. (**c**) TUNEL staining of the liver 4 days after CCl_4_ injection. (**d**) Histological analysis of hCLS using F4/80 immunostaining of the liver 7 days after CCl_4_ injection. (**e**) Sirius red-positive area and representative Sirius red-stained liver sections 7 days after CCl_4_ injection. Scale bars, 100 μm. CV, central veins. (f) Hepatic mRNA expression levels of genes related to fibrogenic factors (*Tgfb1* and *Col1a1*) 7 days after CCl_4_ injection. V, Vehicle. ^#^*p* < 0.05, ^##^*p* < 0.01. Vehicle-treated WT-SD group with olive oil injection, *n* = 10; vehicle-treated MC4R-WD group with CCl_4_ injection, *n* = 10; OCA (10 mg/kg)-treated MC4R-WD group with CCl_4_ injection, *n* = 10.

It is technically difficult to examine the site of action and the causality for pathogenesis in chronic inflammation on a long-term basis. In this regard, we have recently established a novel “inducible NASH model”, in which MC4R-KO mice fed WD for a short term (4 to 6 weeks) were injected with a single low-dose of carbon tetrachloride (CCl_4_, a potent hepatotoxic chemical)^31^. In this novel model, hepatocyte death-triggered inflammation and fibrosis can be sequentially observed during the development of NASH^31^. Thus, we used this model to investigate the effect of OCA on hepatocyte death and the subsequent development of liver fibrosis. OCA (10 mg/kg) or its vehicle (0.5% carboxymethyl cellulose, CMC) was administered daily throughout the experimental period (7 weeks) to the inducible NASH model (Fig. 5a). Treatment with OCA had no effect on body or liver weight (Supplementary Fig. S4). Two days after CCl_4_ injection, p53-regulated gene expression was upregulated, and the number of TUNEL-positive cells was increased 4 days after CCl_4_ injection. These changes were markedly suppressed by treatment with OCA (Fig. 5b,c). Interestingly, treatment with OCA significantly suppressed the number of hCLS and reduced both fibrosis area (Fig. 5d,e) and fibrogenic genes mRNA expression at day 7 after CCl_4_ injection (Fig. 5f). Collectively, these findings suggest that OCA suppresses p53 activation and cell death, thereby preventing the formation of hCLS as well as the development of fibrosis.

### Treatment with OCA ameliorates the progression of NASH

**Figure 6:**
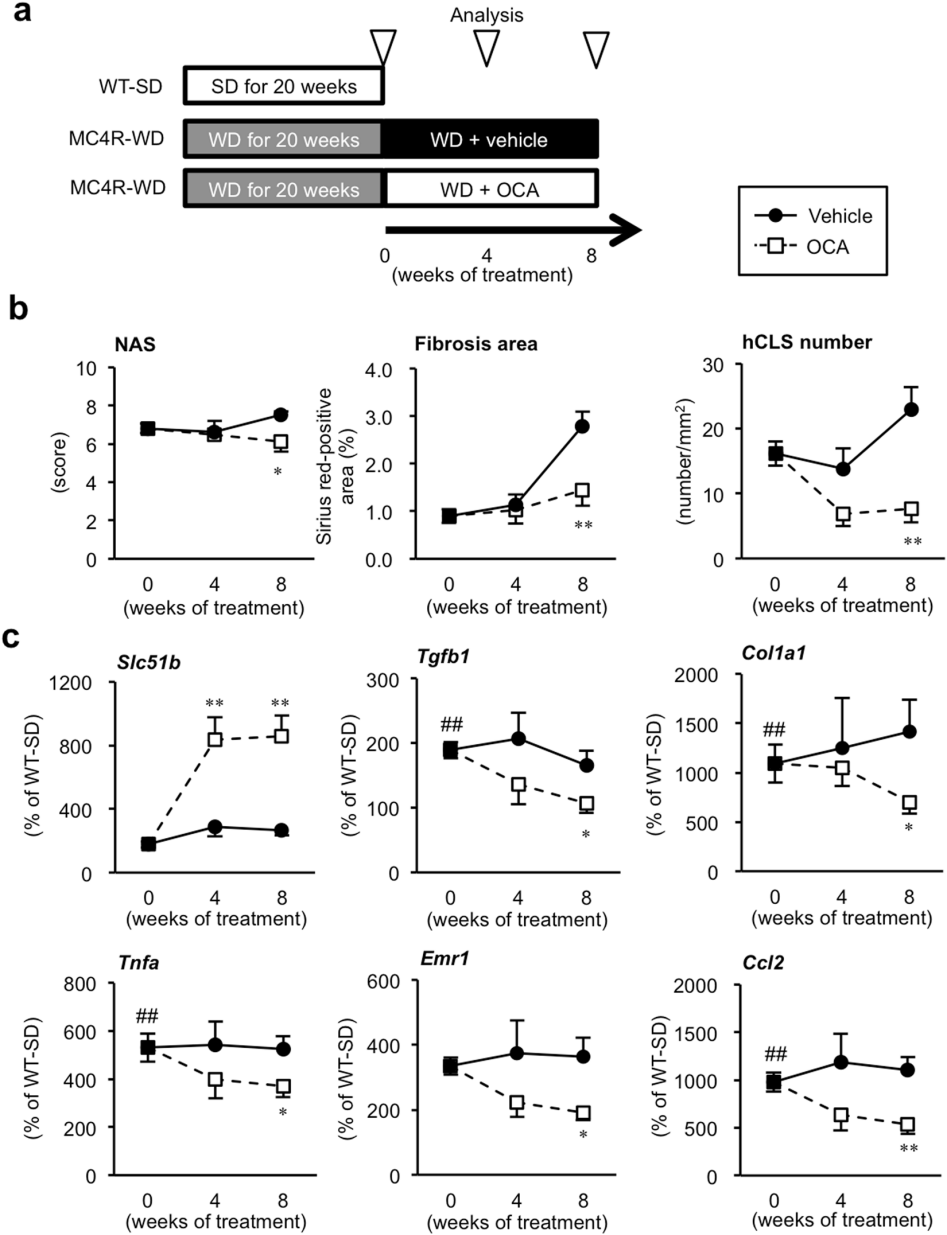
Treatment with OCA ameliorates the progression of NASH in MC4R-KO mice. (**a**) Experimental protocol for evaluation of the effect of OCA on the progression of NASH in MC4R-KO mice. (**b**) Time course of NAS, fibrosis area, and hCLS number in the liver. (**c**) Hepatic mRNA expression levels of FXR-regulated gene (*Slc51b*), inflammatory markers (*Tnfa, Emr1* and *Ccl2*) and fibrogenic factors (*Tgfb1* and *Col1a1*) after 4 and 8 weeks treatment with OCA. ^##^*p* < 0.01 MC4R-WD (pre-treatment) group vs. WT-SD group. ^*^p < 0.05, ^**^p < 0.01 OCA-treated MC4R-WD group vs. the vehicle-treated MC4R-WD group at each time point. Open square, vehicle-treated MC4R-WD group (pretreatment, *n* = 10; 4w and 8w, *n* = 8); closed circle, OCA-treated MC4R-WD group (4w, *n* = 8; 8w *n* = 9). WT-SD, *n* = 7.

In order to evaluate the therapeutic effect of OCA on the progression of NASH, MC4R-KO mice were fed WD for 20 weeks and then received OCA (10 mg/kg) or its vehicle for 4 or 8 weeks (Fig. 6a). Treatment with OCA for 8 weeks significantly decreased serum concentrations of ALT, AST and TC in MC4R-KO mice (Supplementary Table S4), but had no effect on body, liver and adipose tissue weights (Supplementary Fig. S4a). Histological severity and caspase-3/7 activity were suppressed by OCA treatment (Fig. 6b, Supplementary Fig. S5b–e). Furthermore, OCA upregulated mRNA expression of FXR-regulated genes and downregulated that of p53-regulated, inflammatory and fibrogenic genes (Fig. 6c, Supplementary Fig. S5f). These findings suggest that OCA effectively suppresses the progression of liver fibrosis even after MC4R-KO mice developed NASH.

## Discussion

In this study, we showed that treatment with OCA, an FXR agonist, effectively prevents the development of NASH in MC4R-KO mice fed WD. Intriguingly, OCA markedly reduces hCLS formation even after MC4R-KO mice developed NASH, thereby inhibiting the progression to liver fibrosis. As for its mechanism of action, OCA suppressed metabolic stress-induced p53 activation and cell death in hepatocytes, suggesting that these cells are the primary target of FXR agonists. In contrast, several lines of evidence have revealed that FXR agonists display anti-inflammatory and/or anti-fibrotic properties in immune cells, vascular smooth muscle cells, endothelial cells and fibroblasts *in vitro* and *in vivo*^16,17,32,33^. For instance, it is reported that OCA inhibits stellate cell activation and protects against liver fibrosis induced by porcine serum injection or bile duct ligation^18^. On the other hand, we have previously reported that hepatocyte death triggers interaction between stromal cells and fibroblasts during the formation of hCLS, thereby inducing liver fibrosis^24^. Namely, hepatocyte death is considered as an initial event in hCLS formation and the subsequent development of liver fibrosis. Accordingly, it is likely that OCA exerts its beneficial effects on NASH-like liver pathology in MC4R-KO mice, through FXR in hepatocytes. This study sheds light on a novel mechanism, by which FXR agonists protect against NASH.

It is important to discuss the likely mechanism of metabolic stress-induced hepatocyte death and liver fibrosis. Our transcriptome analysis revealed that p53 was the most activated transcription regulator in the livers of MC4R-KO mice. This activation was remarkably suppressed by treatment with OCA. These findings are consistent with recent reports indicating that p53 activation is involved in hepatocyte death and liver fibrosis in both rodent experimental models and human NASH^28–30,34,35^. In this study, treatment with OCA decreased p53 protein levels and suppressed mRNA expression of p53-regulated genes in the livers of MC4R-KO mice. Since OCA treatment does not affect p53 mRNA expression *per se*, post-transcriptional and/or post-translational regulation of p53 may contribute to FXR-mediated hepato-protective effects. In this regard, it is known that SHP physically interacts with MDM2 to increase MDM2 protein stability, which in turn augments p53 ubiquitination^36^; *i.e*. FXR stabilizes MDM2 protein through induction of *Shp* expression. Moreover p53 acetylation, particularly in the C-terminal region, inhibits the recruitment of MDM2 to p53, thereby abrogating MDM2-mediated repression of p53^37,38^. For the next step, it is interesting to know how OCA regulates p53 modifications in NASH liver. In addition, it is known that metabolic stress- and proinflammatory cytokine-induced production of ROS potently increases p53 activity^39,40^. In line with the previous report showing that FXR suppresses oxidative stress^41,42^, OCA treatment effectively suppressed ROS production in the liver of MC4R-KO mice. Taken together, these findings suggest that OCA potently suppresses metabolic stress-induced p53 stability and/or ROS production in hepatocytes, thereby inhibiting p53 activation and hepatocyte death during the development of NASH in MC4R-KO mice.

The metabolic effects of OCA in MC4R-KO mice should be discussed further, although OCA treatment showed only marginal effect on body, liver, and adipose tissue weights in MC4R-KO mice. For instance, it is known that bile acids and cholesterol play a role in obesity-induced chronic inflammation and tumorigenesis in the liver^43–45^. Treatment with OCA markedly suppressed the otherwise increased levels of total bile acids in the livers of MC4R-KO mice, whereas it had no effect on hepatic levels of cytotoxic secondary bile acids (*e.g*. deoxycholic acid and taurodeoxycholic acid) metabolized by gut microbiota. On the other hand, FXR agonists could exert their cholesterol-lowering effects through several mechanisms, including stimulation of bile acid secretion from hepatocytes and inhibition of bile acid absorption in the intestine^46,47^. Recent evidence also suggests that increased molar ratio of cholesterol-phospholipids in certain cells reduces mitochondrial glutathione levels, which may increase oxidative stress and cause cell death^48^. Indeed, hepatic and serum levels of cholesterol were decreased by treatment with OCA in MC4R-KO mice, which may be the source of hepato-protective effects of OCA. Since serum cholesterol levels were rather increased in the FLINT study^20^, there may be differences in serum lipids regulation among species. Accordingly, it is important to further examine how the metabolic effects of OCA contribute to hepatocyte death, hCLS formation and liver fibrosis in NASH.

Consistent with the FLINT study, OCA improved ballooning degeneration, lobular inflammation and interstitial fibrosis in the livers of MC4R-KO mice. In this regard, this study highlights the effects of OCA on metabolic stress-induced hepatocyte injury and the subsequent inflammatory and fibrogenic responses. This notion is supported by our experiment in the inducible NASH model, in which OCA effectively suppressed CCl_4_-induced p53 activation and hepatocyte injury without affecting hepatic steatosis. Another clinical implication of this study is that OCA is effective in the therapeutic protocol, since it is currently difficult to preemptively treat NASH, largely due to lack of clinically useful biomarkers. Our findings show that serum concentrations of soluble VCAM-1, a novel biomarker for human NASH^27^, can reflect disease conditions in MC4R-KO mice. For future investigation, it would be interesting to examine how soluble VCAM-1 is released in NASH and how sensitive and selective this biomarker is. There are currently a number of FXR agonists with distinct properties under development. However, appropriate biomarkers as well as animal models recapitulating human NASH are needed to accelerate the development of novel therapeutic strategies for NASH.

In conclusion, we have shown in this study that OCA effectively prevents the development and progression of NASH in a unique animal model with obesity and insulin resistance. Among various cell types expressing FXR, our findings indicate that OCA acts mainly on hepatocytes, where it inhibits metabolic stress-induced p53 activation and hepatocyte death, thereby suppressing hCLS formation and interstitial fibrosis. Collectively, the results of this study highlight the novel anti-fibrotic effects of OCA, suggesting its therapeutic use for human NASH.

## Materials and Methods

### Materials

OCA was synthesized in Sumitomo Dainippon Pharma Co., Ltd (Osaka, Japan) laboratories and suspended in 0.5% CMC (vehicle). All reagents were purchased from Sigma (St. Louis, MO, USA) or Nacalai Tesque (Kyoto, Japan) unless otherwise noted.

### Animals

MC4R-KO mice with C57BL/6J background were kindly provided by Dr. Joel K. Elmquist (University of Texas Southwestern Medical Center)^23^, and age-matched C57BL/6J WT mice were purchased from CLEA Japan (Tokyo, Japan). The animals were housed in a temperature-, humidity- and light-controlled animal room (12-h light and 12-h dark cycle), and allowed free access to water and SD (CE-2; CLEA Japan). In the first experiment, eight week-old male MC4R-KO mice were fed WD (D12079B; 468 kcal/100 g, 41% energy as fat, 34.0% sucrose, 0.21% cholesterol; Research Diets, New Brunswick, NJ) instead of the SD for 24 weeks. OCA (3 or 10 mg/kg) or 0.5% CMC was administered daily by gavage to MC4R-KO mice throughout the experiment (24 weeks) to evaluate its preventive effect on NASH. Eight-week-old control male WT mice were fed SD throughout the experimental period and administrated 0.5% CMC daily. To evaluate the effect of OCA on the progression of NASH, we initiated another experiment where eight-week-old MC4R-KO mice were fed WD for 20 weeks and control WT mice were fed SD for the same period. OCA (10 mg/kg) was administered daily for 4 or 8 weeks to mice that have developed NASH following WD diet. At the end of all experiments, the mice were sacrificed, when fed *ad libitum*, under intraperitoneal pentobarbital anesthesia (30 mg/kg). All animal experiments were conducted in accordance to the guidelines for the care and use of laboratory animals of Tokyo Medical and Dental University. The protocols were approved by Tokyo Medical and Dental University Committee on Animal Research (No. 2015–008 C, No. 0170186 A).

### Blood analysis

BG levels were measured by a blood glucose test meter (Glutest PRO R; Sanwa-Kagaku, Nagoya Japan). Serum concentrations of ALT, AST, TC, TG and NEFA were measured by the respective standard enzymatic assays using a Clinical Biochemistry Analyzer (JCA-BM1650, JEOL, Tokyo Japan). Serum concentrations of insulin, soluble VCAM-1 and collagen type IV were measured using Revis Insulin ELISA kit (Shibayagi, Gunma, Japan), sVCAM-1/CD106 Quantikine ELISA Kit (R&D Systems, Minneapolis, MN, USA) and Mouse Collagen IV ELISA kit (LifeSpan BioSciences, Seattle, WA, USA), respectively. The amounts of serum, liver and ileum BA were measured by LC-MS-MS in the Junshin Clinic Bile Acid Institute (Tokyo, Japan).

### Histological analysis

Liver samples were fixed with neutral-buffered formalin, embedded in paraffin and cut into 4 μm thick sections that were stained with Hematoxylin and eosin and Sirius red^22^. F4/80-positive macrophages were detected immunohistochemically using a rat monoclonal anti-mouse F4/80 antibody (MCA497GA, Serotec, UK). Apoptotic cells were detected by TUNEL assay using an Apop-Tag Plus Peroxidase *In Situ* Apoptosis Detection Kit (Millipore, Billerica, MA, USA), and anti-Caspase-3 antibody (ab13847, Abcam, Cambridge, UK). Sirius red-positive area was measured using the software WinROOF (Mitani, Chiba, Japan). TUNEL-positive cells and hCLS were counted in the whole area of each section and expressed as the mean number/mm^2^. According to the NASH clinical research network scoring system, each score for steatosis, inflammation, and hepatocyte ballooning was evaluated by two investigators^49^. Briefly, the degree of steatosis, inflammation, and hepatocyte ballooning was scored using hematoxylin & eosin-stained liver sections. Each variable was graded from zero to three, and the sum of the scores was considered as NAFLD activity score. Fibrosis was staged from zero to three using Sirius red-stained sections.

### Caspase-3/7 activity assay

Hepatic caspase-3/7 was measured using Caspase-Glo 3/7 assay (Promega, Madison, WI, USA) according to the manufacturer’s instruction. Briefly, tissue was homogenized in hypotonic extraction buffer (25 mM HEPES (pH 7.5), 5 mM MgCl2, 1 mM EGTA) with protease inhibitors (Sigma). After centrifugation at 15,000 × g, 15 min, supernatants were collected and determined the protein concentrations. Equal volume of protein samples and assay reagents were incubated at room temperature for 1 hour, and luminescence of each sample was measured using luminometer.

### Evaluation of Oxidative stress

The liver was lysed in a RIPA buffer (0.1% SDS, 1% Nonidet P-40, 5 mM EDTA, 150 mM NaCl, 50 mM Tris pH 7.6) supplemented with protease inhibitor cocktail (Sigma). 4-HNE was determined using the OxiSelect HNE Adduct ELISA Kit (Cell Biolabs, San Diego, CA, USA).

### Inducible NASH model

MC4R-KO mice fed WD for 6 weeks were given a single intraperitoneal injection of CCl_4_ (WAKO, Osaka, Japan) diluted 1:20 in olive oil (vehicle) to the dose of 0.2 ml/kg. The mice were then kept on WD after CCl_4_ injection and sacrificed at pre-determined time points. MC4R-KO mice were treated by gavage once daily with OCA at 10 mg/kg or the vehicle (0.5% CMC) throughout the experimental period.

### Isolation of liver cells

Hepatocytes and NPCs were isolated from WT and MC4R-KO mice by a 2-step protocol (collagenase digestion and Percoll gradient) as previously described^25^. The mice were anesthetized, and the liver was perfused via the portal vein with Hank’s Balanced Salt Solution containing 0.5 mM ethylene glycol tetraacetic acid, followed by Dulbecco’s Modified Eagle Medium (DMEM) containing 75 mg/dL collagenase type IV. The excised liver was immediately diced into small pieces, and filtered through a 100 μm cell strainer to remove debris. The filtered cells were suspended in DMEM supplemented 10% fetal bovine serum (Biowest, Nuaille, France), and primary hepatocytes were separated from the NPCs by centrifugation at 50 × g for 3 minutes. Hepatocytes were purified using density gradient centrifugation (45% Percoll) at 100 × g for 7 minutes. After hepatocytes removal, NPCs were resuspended in 30% Percoll and centrifuged at 600 × g for 15 minutes to remove cell debris. Red blood cells were lysed with Ammonium-Chloride-Potassium buffer and NPCs were washed twice with phosphate buffered saline.

### Quantitative Real-Time PCR

Total RNA was extracted from the liver, the isolated hepatocytes and NPCs using Sepasol reagent. Quantitative real-time PCR was performed with StepOnePlus Real-time PCR System using Fast SYBR Green Master Mix Reagent (Applied Biosystems, Foster City, CA, USA) as described previously^26^. The primers used in this study are listed in Supplementary Table S1. Levels of mRNA were normalized to those of 36B4 mRNA.

### Microarray

Microarray analysis was performed using SurePrint G3 Mouse GE 8 × 60 K microarrays (Agilent Technologies, Palo Alto, CA, USA) according to the manufacturer’s instructions. Differentially expressed genes were selected based on fold change and *p* value (fold change >1.5, *p* > 0.05). Data were analyzed by QIAGEN’s Ingenuity® Pathway Analysis (IPA®, QIAGEN Red wood City, www.qiagen.com/ingenuity).

### Western blotting

The liver was lysed in a RIPA buffer supplemented with Halt Protease and Phosphatase Inhibitor Cocktail (Thermo Fisher Scientific, San Jose, CA, USA). Proteins were separated by SDS-PAGE and immunoblotted with a p53 (8592, Cell Signaling Technology, Beverly, MA, USA) and α-Tubulin antibody (2144, Cell Signaling Technology). Immunoblots were detected and analyzed with ECL Prime Western Blotting Detection Reagent and ImageQuant LAS 4000 mini (GE Healthcare, Little Chalfont, UK).

### Hepatic triglyceride and cholesterol levels

Total lipids in the liver were extracted with ice-cold 2:1 (vol/vol) chloroform/methanol. Hepatic triglyceride (TG) and cholesterol concentrations were determined using TG E-test Wako and cholesterol E-test WAKO (Wako), respectively.

### Statistical analysis

Data are presented as the mean ± SE. *p* values < 0.05 were considered as statistically significant. Statistical analysis was performed using analysis of variance followed by Dunnetts’ multiple comparison test. Differences between two groups were compared using unpaired Student *t*-test.

## Electronic supplementary material

Supplementary information
