# Supplementary material for: Obeticholic acid protects against hepatocyte death and liver fibrosis in a murine model of nonalcoholic steatohepatitis
Source: Sci Rep. 2018 May 25;8:8157. doi: 10.1038/s41598-018-26383-8 (PMC5970222; doi:10.1038/s41598-018-26383-8)
Supplement: Supplementary file 1 — Supplementary information [file 41598_2018_26383_MOESM1_ESM.pdf]

Obeticholic acid protects against hepatocyte death and liver fibrosis in a murine model of nonalcoholic steatohepatitis

Authors:

Toshihiro Goto[1,2], Michiko Itoh[3], Takayoshi Suganami[4]*, Sayaka Kanai[5], Ibuki Shirakawa[3], Takeru Sakai[1], Masahiro Asakawa[1], Toshihiro Yoneyama[6], Toshihiro Kai[6], Yoshihiro Ogawa[1,4,5,7,8]*

Institutional affiliation:

[1]Department of Molecular Endocrinology and Metabolism, Graduate School of Medical and Dental Sciences, Tokyo Medical and Dental University, Tokyo, Japan; [2]Specialty Medicine Group Drug Development Research Laboratories Sumitomo Dainippon Pharma. Co., Ltd., Osaka, Japan; [3]Department of Organ Network and Metabolism, Graduate School of Medical and Dental Sciences, Tokyo Medical and Dental University, Tokyo, Japan; [4]Department of Molecular Medicine and Metabolism, Research Institute of Environmental Medicine, Nagoya University, Nagoya, Japan; [5]Department of Molecular and Cellular Metabolism, Graduate School of Medical and Dental Sciences, Tokyo Medical and Dental University, Tokyo, Japan; [6]Omics Group, Genomic Science Laboratories, Sumitomo Dainippon Pharma. Co., Ltd., Osaka, Japan; [7]Department of Medicine and Bioregulatory Science, Graduate School of Medical Sciences, Kyushu University, Fukuoka, Japan; [8]Japan Agency for Medical Research and Development, CREST, Tokyo, Japan. [9]T.G. and M.I. contributed equally to this work and share the first authorship.

Supplementary materials

Supplementary method

Western blotting

Nuclear proteins were extracted using NE-PER Nuclear and Cytoplasmic Extraction Reagents (Thermo Fisher Scientific, San Jose, CA, USA) supplemented with Halt Protease and Phosphatase Inhibitor Cocktail and sodium butyrate. Proteins were separated by SDS-PAGE and immunoblotted with antibodies against MDM2 (ab16895, abcam), phospho-p53 (Ser15) (9284, Cell Signaling) and acetyl-p53 (Lys379) (2570, Cell Signaling), and Lamin A/C (sc-20168, Santa Cruz).

Supplementary Table S1. Primers used in the present study

Genes	Primers	Primers
Abcb11	Forward	GTCTGACTCAGTGATTCTTCGC
	Reverse	GAGCAATGCGCACACACTTC
Apaf1	Forward	CTCTAGATGAAGCCATGTCG
	Reverse	AAGGATGGAAAGGTCTGTGT
Ccl2	Forward	CCACTCACCTGCTGCTACTCAT
	Reverse	TGGTGATCCTCTTGTAGCTCTCC
Col1a1	Forward	CCTCAGGGTATTGCTGGACAAC
	Reverse	ACCACTTGATCCAGAAGGACCTT
Col4a1	Forward	TCCATGGCACCCATCTCTG
	Reverse	CTTCACAAACCGCACACCTG
Ctgf	Forward	CTTCTGCGATTTCGGCTCC
	Reverse	TACACCGACCCACCGAAGA
Cyp7a1	Forward	CTGATCCGTCTACGCATGTTTC
	Reverse	CAGGAATGGTGTTTGCTTGAGA
Cyp8b1	Forward	ACGCTTCCTCTATCGCCTGAA
	Reverse	GTGCCTCAGAGCCAGAGGAT
Emr1	Forward	CTTTGGCTATGGGCTTCCAGT
	Reverse	GCAAGGAGGACAGAGTTTATCGTG
Mdm2	Forward	AAGGAGGAAACGCAGGACAA
	Reverse	TCTTGCCGTGAACAATGCA
Nr0b2	Forward	AAGGGCACGATCCTCTTCAA
	Reverse	GTACCAGGGCTCCAAGACT
Nr1h4	Forward	AGGCCATGTTTCTTCGTTCG
	Reverse	CATGACCGGCAGGAAGTTTC

P21	Forward	AACATCTCAGGGCCGAAAAC
	Reverse	CTGCGCTTGGAGTGATAGAA
Slc51b	Forward	GACAAGCATGTTCCTCCTGAG
	Reverse	GATGCAGGTCTTCTGGTGTTTC
Srebf1c	Forward	AGCTGTCGGGGTAGCGTCTG
	Reverse	GAGAGTTGGCACCTGGGCTG
Tgfb1	Forward	CCTGAGTGGCTGTCTTTTGACG
	Reverse	AGTGAGCGCTGAATCGAAAGC
Timp1	Forward	CATCACGGGCCGCCTA
	Reverse	AAGCTGCAGGCACTGATGTG
Tnfa	Forward	ACCCTCACACTCAGATCATCTTC
	Reverse	TGGTGGTTTGCTACGACGT
Tnfs10b	Forward	TGCTGCTCAAGTGGCGC
	Reverse	GGCATCCAGCAGATGGTTG
Tp53	Forward	CTCAAAAAACTTACCAGGGC
	Reverse	CACCACGCTGTGGCGAAAAGTCTG
Vcam1	Forward	TCACAATTAAGAAGTTTAACACTTGATGTAA
	Reverse	GAGTGCAAGGAGTTCGGGC
36B4	Forward	GGCCCTGCACTCTCGCTTTC
	Reverse	TGCCAGGACGCGCTTGT

Supplementary Table S2. Effect of OCA treatment on total bile acid in MC4R-KO fed WD for 24 weeks.

	WT-SD	MC4R-WD	
	Vehicle	Vehicle	OCA
Serum bile acid (μmol/L)	3.69	21.25	2.33
Liver bile acid (μmol/g tissue)	0.16	0.34	0.11
Ileum bile acid (μmol/g tissue)	0.62	2.13	0.73

Serum and tissue bile acid levels were measured using samples pooled from 6 mice for WT-SD, 10 mice for vehicle-treated MC4R-WD, 9 mice for OCA-treated MC4R-WD.

Supplementary Table S3. Effect of OCA on hepatic bile acid composition (%) in MC4R-KO fed WD for 24 weeks.

	WT-SD	MC4R-WD	
	Vehicle	Vehicle	OCA
TCA	30.4	40.2	10.1
CA	0.8	0.4	0.0
TCDCA	2.4	3.2	6.5
CDCA	0.1	0.0	0.0
GUDCA	0.0	0.0	0.0
TUDCA	2.3	3.7	10.7
UDCA	0.2	0.1	0.8
TDCA	4.7	0.7	0.0
DCA	0.0	0.0	0.0
THCA	2.3	0.7	0.9
HCA	0.0	0.0	0.0
TLCA	0.0	0.0	0.0
LCA	0.0	0.0	0.0
T-α-MCA	3.0	5.9	7.2
α-MCA	0.7	0.4	0.8
T-β-MCA	22.8	32.2	40.0
β-MCA	12.7	6.1	12.0
T-ω-MCA	11.1	5.2	7.7
ω-MCA	3.5	0.6	1.4

CA, cholic acid; TCA, tauro-CA; CDCA, chenodeoxycholic acid; TCDCA, tauro-CDCA;

UDCA, ursodeoxycholic acid; GUDCA, glycol-UDCA; TUDCA, tauro-UDCA; DCA, deoxycholic acid; TDCA, tauro-DCA; HCA, hyocholic acid; THCA, tauro-HCA; LCA, lithocholic acid; TLCA, tauro-LCA; α/β/ω-MCA, α/β/ω-muricholic acid; T-α/β/ω-MCA, tauro-α/β/ω-MCA. Hepatic bile acid composition was measured using samples pooled from 6 mice for WT-SD, 10 mice for vehicle-treated MC4R-WD, 9 mice for OCA-treated MC4R-WD.

Supplementary Table S4. Serological parameters of MC4R-KO treated with OCA for 4 or 8 weeks after the development of NASH.

	MC4R-WD				
	-	Vehicle	OCA	Vehicle	OCA
Weeks	0	4	4	8	8
BG (*ad lib*, mg/dL)	149.6 ± 9.4	150.0 ± 7.0	167.9 ± 8.6	152.1 ± 12.4	179.2 ± 9.2
TC (mg/dL)	320.6 ± 11.3##	328.0 ± 27.4	263.0 ± 11.0*	336.3 ± 8.8	270.9 ± 19.3**
TG (mg/dL)	60.4 ± 7.1	78.8 ± 11.4	56.5 ± 7.2	56.3 ± 4.8	52.7 ± 6.3
NEFA (mEq/L)	0.96 ± 0.74	0.81 ± 0.40	0.66 ± 0.32	0.72 ± 0.62	0.73 ± 0.50
ALT (U/L)	344.6 ± 26.4##	499.5 ± 104.5	323.8 ± 52.2	700.0 ± 92.2	323.6 ± 55.0**
AST (U/L)	417.0 ± 45.3##	426.5 ± 53.6	329.3 ± 62.1	580.0 ± 63.2	300.9 ± 33.6**
VCAM-1 (μg/mL)	1.37 ± 0.05##	1.43 ± 0.06	1.21 ± 0.03**	1.38 ± 0.07	1.19 ± 0.06

VCAM-1, vascular cell adhesion molecule-1; -, no treatment. Data are expressed as the mean ± SE. n = 5-10. #$p < 0.05$, ##$p < 0.01$ MC4R-WD (pre-treatment) group vs. WT-SD group, *$p < 0.05$, **$p < 0.01$ vs. MC4R-WD group treated with the vehicle.

Supplementary Figures

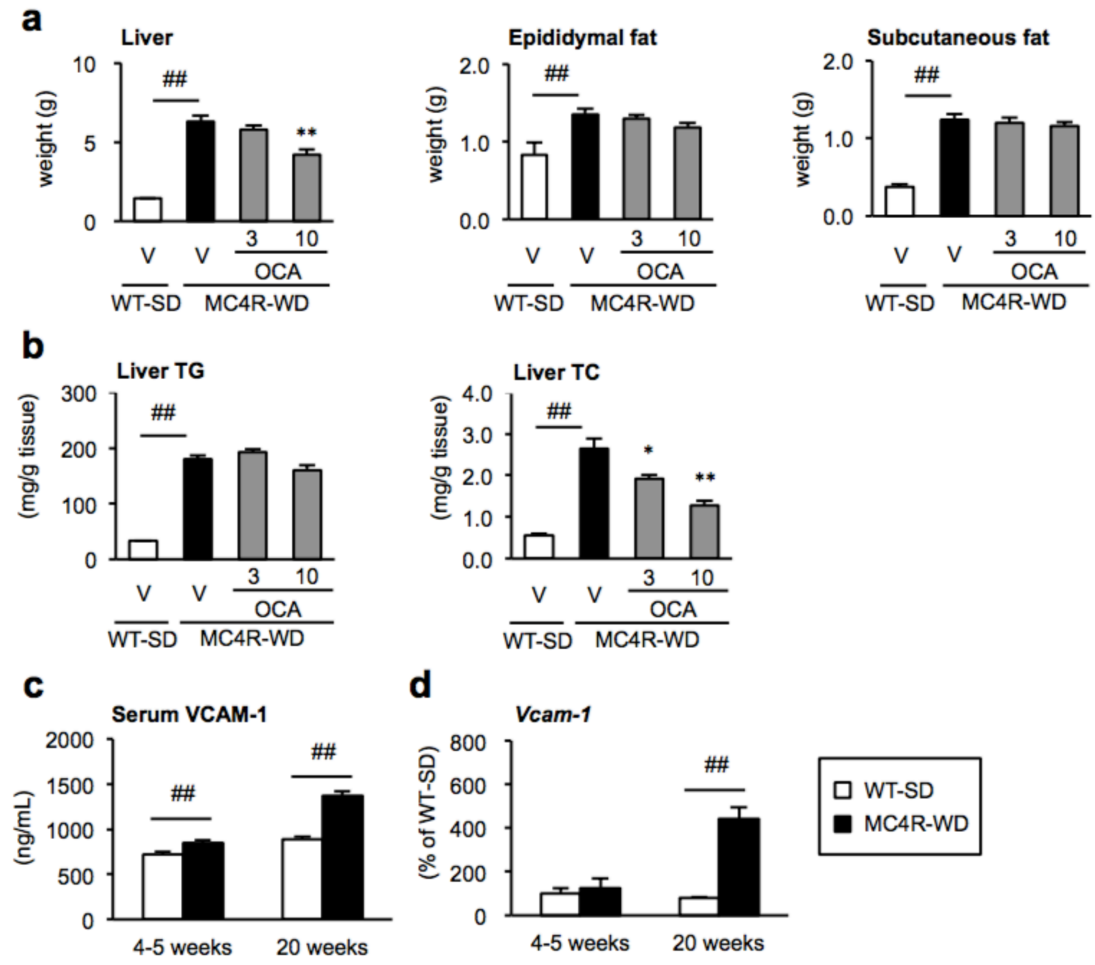

Supplementary Figure S1. Effect of OCA on tissue weight and liver triglyceride and cholesterol levels in MC4R-KO mice.

(a) Body weight and liver, subcutaneous and epididymal adipose tissue weights in MC4R-KO and WT mice. (b) Liver triglyceride (TG) and total cholesterol (TC) levels at 24 weeks. (c) Serum soluble vascular cell adhesion molecule-1 (VCAM-1) concentrations and (d) hepatic mRNA expression levels of VCAM-1 in MC4R-KO mice fed WD for 4-5 weeks or 20 weeks compared to age-matched WT mice fed SD. V, Vehicle. $^{\#\#}p < 0.01$; $^{*}p < 0.05$, $^{**}p < 0.01$ vs. the vehicle-treated MC4R-WD group.

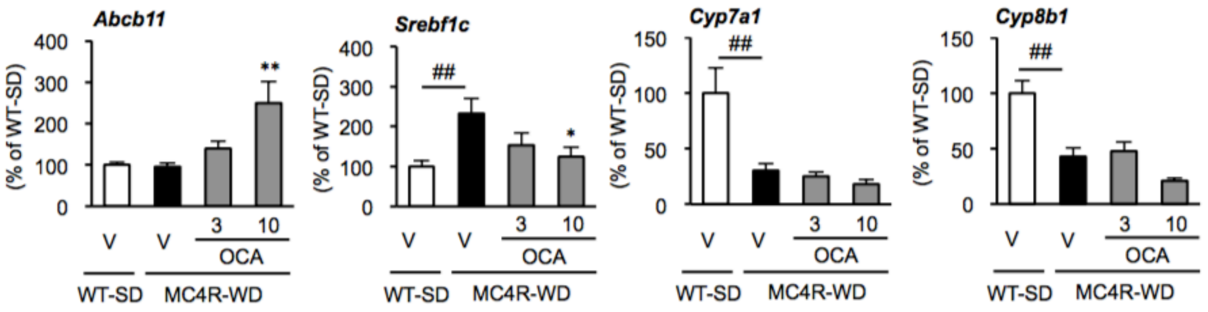

Supplementary Figure S2. Hepatic mRNA expression of FXR-regulated genes after 24-week OCA treatment.

Hepatic mRNA expression levels of ATP-binding cassette subfamily B member 11 (*Abcb11*), sterol regulatory element binding protein 1c (*Srebf1c*), cytochrome P450 7A1 (*Cyp7a1*) and cytochrome P450 8b1 (*Cyp8b1*) after treatment with OCA for 24 weeks. $^{\#\#}p < 0.01$; $^{**}p < 0.01$ vs. vehicle-treated MC4R-WD group. Vehicle-treated WT-SD group, $n = 7$; vehicle-treated MC4R-WD group, $n = 10$; 3 and 10 mg/kg of OCA-treated MC4R-WD group, $n = 10$ and $n = 9$, respectively.

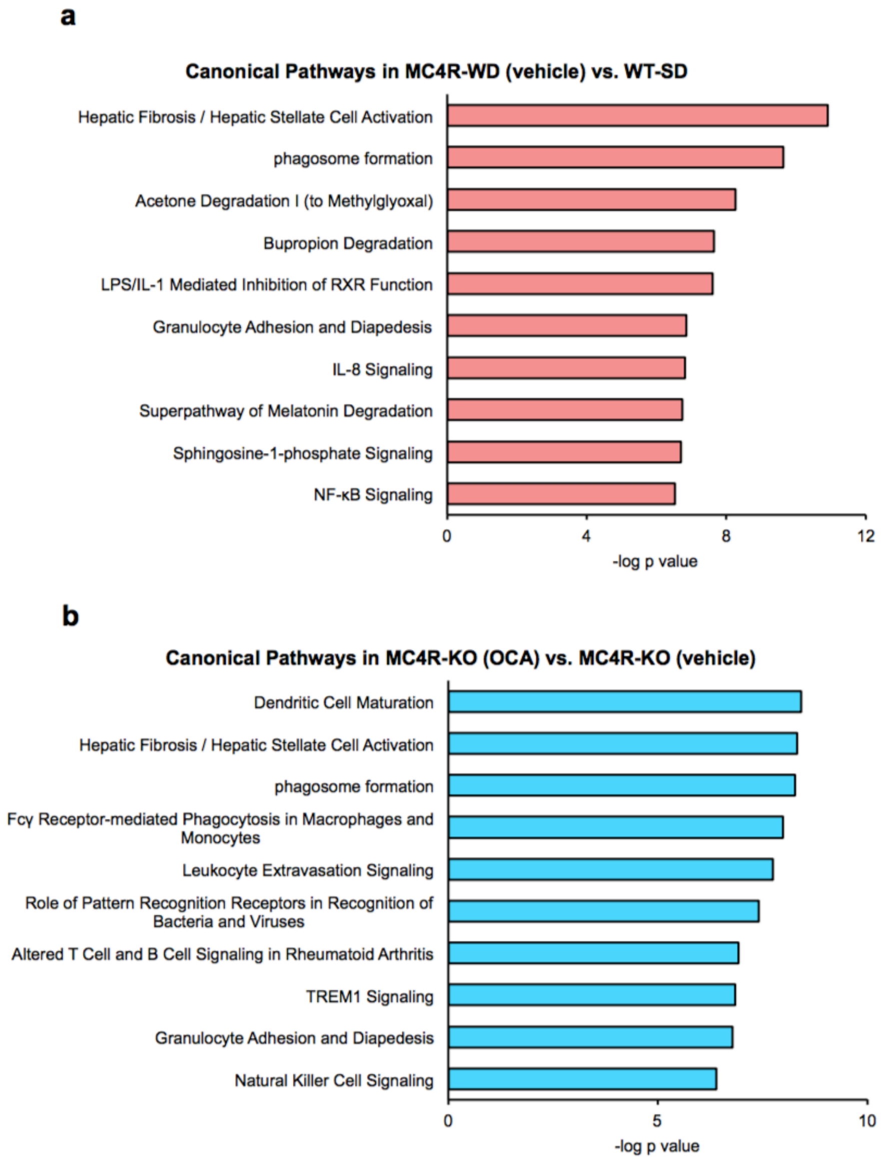

Supplementary Figure S3. Effect of OCA on canonical pathways in NASH.

Ingenuity Pathway Analysis of top 10 canonical pathways for (a) vehicle-treated WT-SD group vs. vehicle-treated MC4R-WD group and (b) vehicle-treated MC4R-WD group vs. OCA-treated MC4R-WD group.

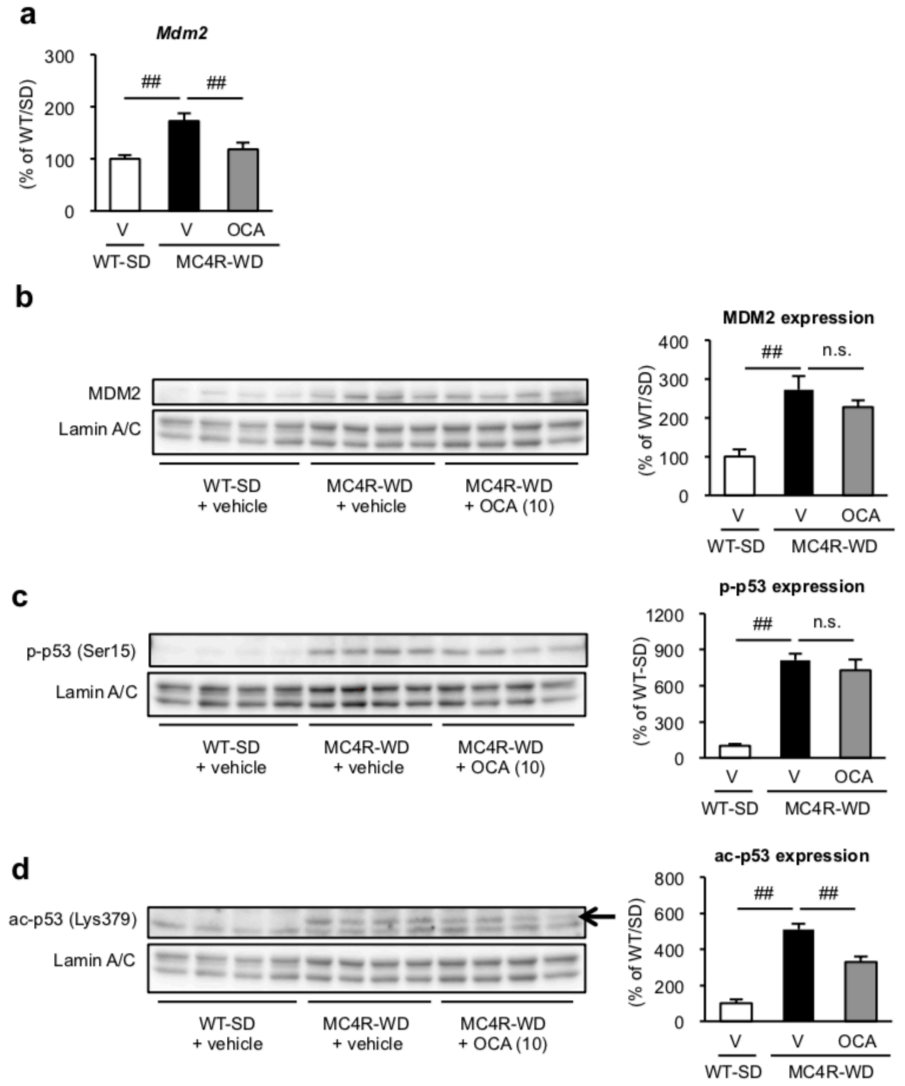

Supplementary Figure S4. Effect of OCA on modification status of p53 in the liver.

(a) Hepatic mRNA expression levels of *Mdm2*. Vehicle-treated WT-SD group, n = 7; vehicle-treated MC4R-WD group, n = 10; 10 mg/kg OCA-treated MC4R-WD group, n = 9. Nuclear protein levels of MDM2 (b), phospho-p53 (Ser15) (c) and acetyl-p53 (Lys379) (d). n = 4. ## $p < 0.01$, n.s., not significant.

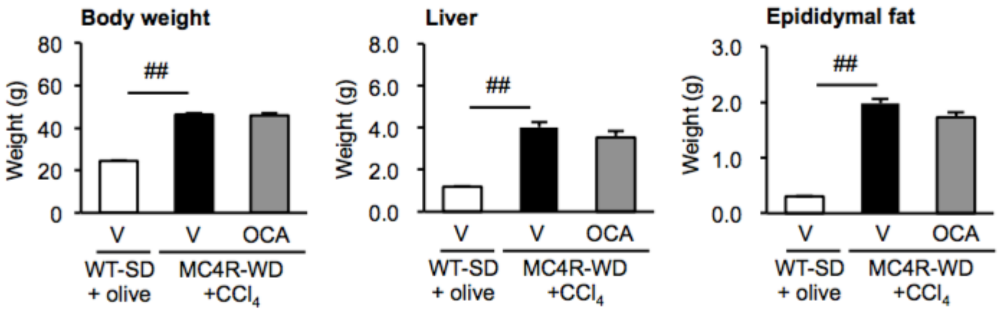

Supplementary Figure S5. Effect of OCA on body and tissue weights in the inducible NASH model.

Body, liver and epididymal fat weights 7 days after CCl_4 injection. V, Vehicle. $^{\#}p < 0.05$, $^{\#\#}p < 0.01$. Vehicle-treated WT-SD group with olive oil injection, $n = 10$; vehicle-treated MC4R-WD group with CCl_4 injection, $n = 10$; OCA (10 mg/kg)-treated MC4R-WD group with CCl_4 injection, $n = 10$.

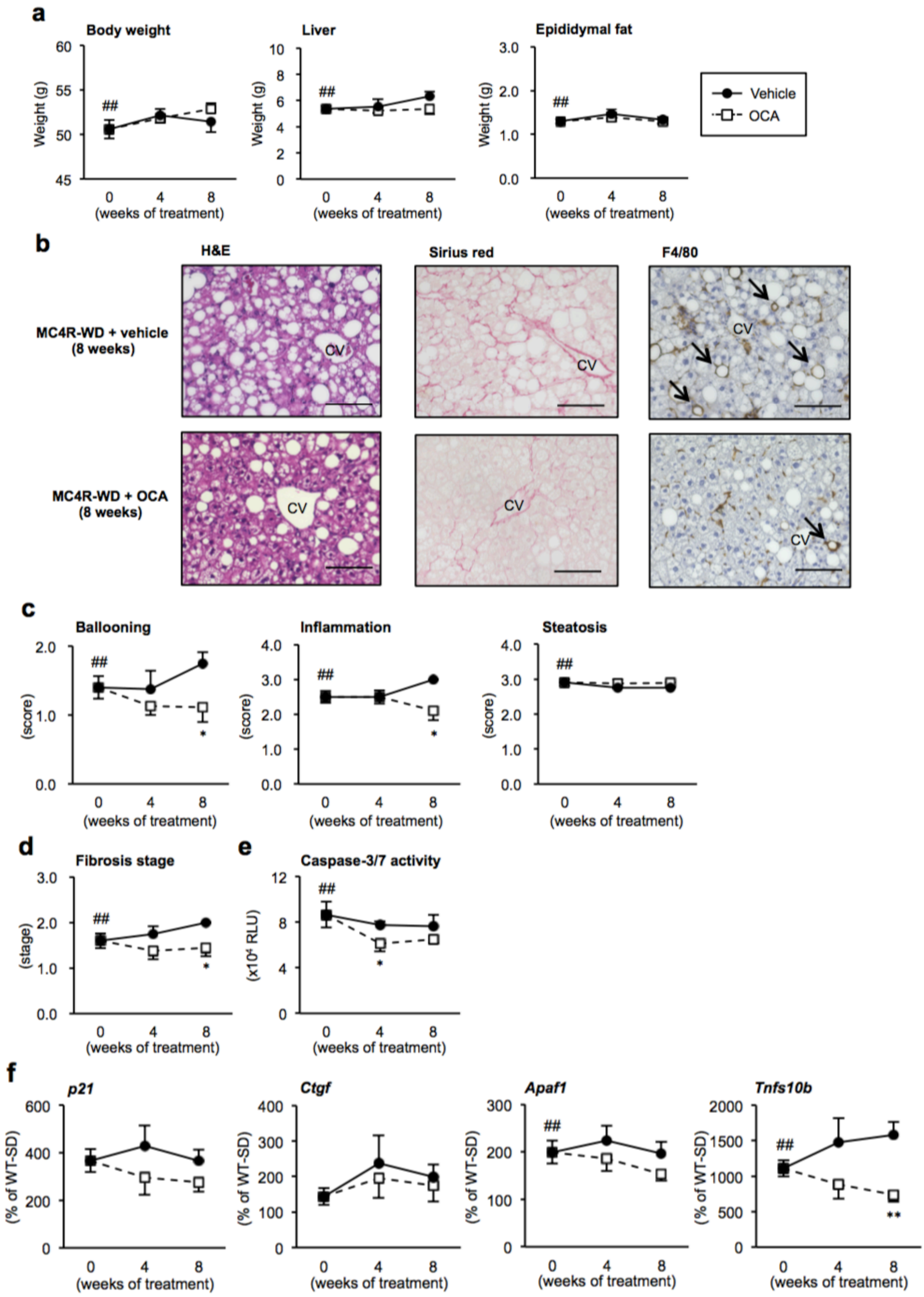
a
Body weight
Liver
Epididymal fat
Weight (g)
(weeks of treatment)
Vehicle
OCA
##
b
H&E
Sirius red
F4/80
MC4R-WD + vehicle (8 weeks)
MC4R-WD + OCA (8 weeks)
CV
c
Ballooning
Inflammation
Steatosis
(score)
d
Fibrosis stage
(stage)
e
Caspase-3/7 activity
(x10⁴ RLU)
f
p21
Ctgf
Apaf1
Tnfs10b
(% of WT-SD)

Supplementary Figure S6. Effect of OCA on the progression of NASH in MC4R-KO mice.

(a) Time course of body, liver and epididymal fat weights. (b) Representative images of Hematoxylin and eosin, Sirius red staining, and F4/80 immunostaining of the livers from WD-fed MC4R-KO mice treated with OCA for 8 weeks. Arrows, hCLS. Scale bars, 100 µm. CV, central veins. Time course of ballooning, inflammation and steatosis scores (c), and fibrosis stage in the liver (d). Caspase-3/7 activity in the liver (e). (f) Time course of hepatic mRNA expression levels of *p21, Ctgf, Apaf1* and *Tnfs10b* after 4 or 8 weeks treatment with OCA. $^{\#\#}p < 0.01$ MC4R-WD (pre-treatment) vs. WT-SD. $^{*}p < 0.05$, $^{**}p < 0.01$ OCA-treated MC4R-WD group vs. vehicle-treated MC4R-WD group at each point. Open square, vehicle-treated MC4R-WD group (pretreatment, $n = 10$; 4w and 8w, $n = 8$); closed circle, OCA-treated MC4R-WD group (4w, $n = 8$; 8w $n = 9$). WT-SD, $n = 7$.

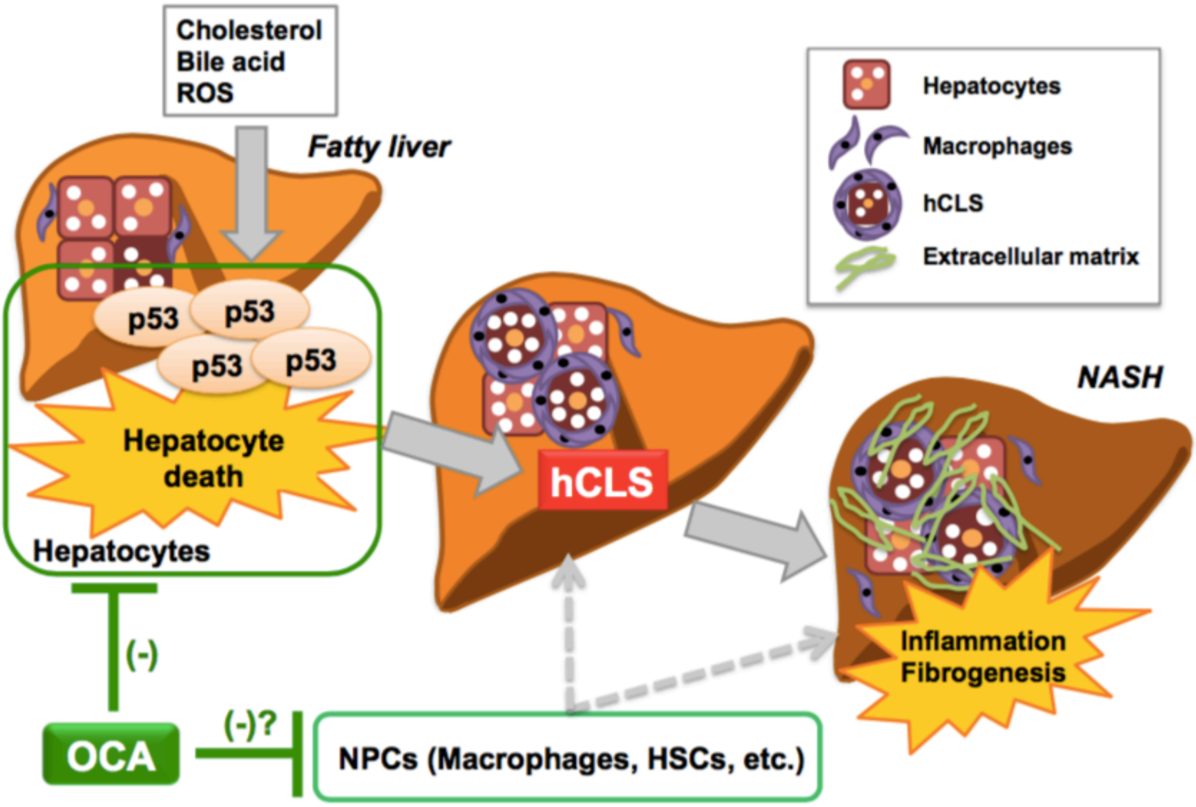

Supplementary Figure S7. Graphical summary

hCLS, hepatic crown-like structure; HSC, hepatic stellate cell; NASH, non-alcoholic steatohepatitis; NPC, non-parenchymal cell; OCA, obeticholic acid; ROS, reactive oxygen species.

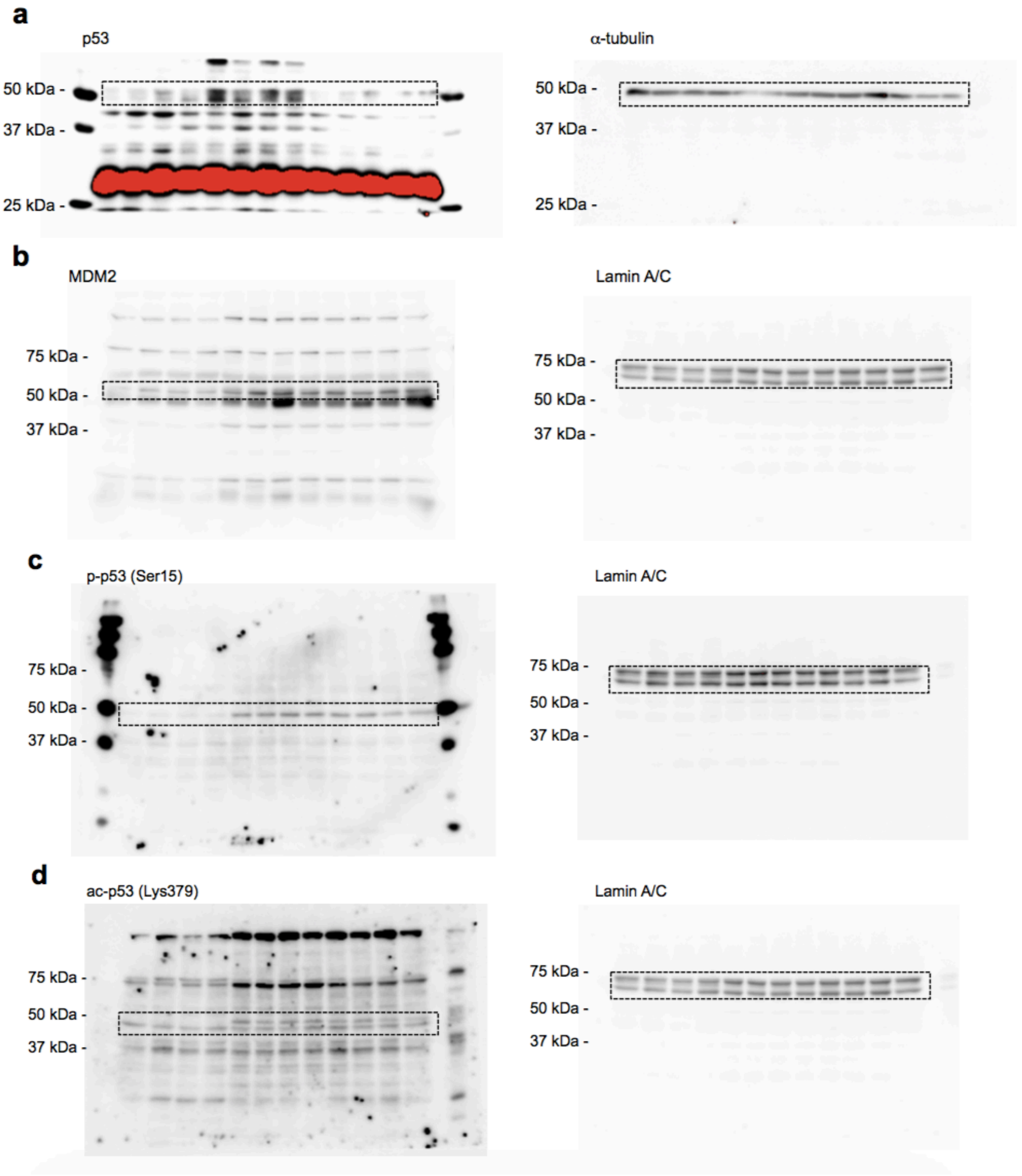

Supplementary Figure S8. Uncropped Western blots for Fig. 3c and Supplmentary Fig. S4.

Boxes indicate cropped areas.
